# Loss of DOCK2 potentiates Inflammatory Bowel Disease–associated colorectal cancer via immune dysfunction and IFNγ induction of IDO1 expression

**DOI:** 10.1038/s41388-024-03135-9

**Published:** 2024-09-07

**Authors:** Antonia M. D. Churchhouse, Caroline V. Billard, Toshiyasu Suzuki, Sebastian Ö. G. Pohl, Nora J. Doleschall, Kevin Donnelly, Colin Nixon, Mark J. Arends, Shahida Din, Kathryn Kirkwood, Jair Marques Junior, Alex Von Kriegsheim, Seth B. Coffelt, Kevin B. Myant

**Affiliations:** 1 Institute of Genetics and Cancer, The University of Edinburgh, Western General Hospital Campus, Edinburgh, UK; 2 Cancer Research UK Scotland Institute, Garscube Estate, Glasgow, UK; 3 School of Cancer Sciences, University of Glasgow, Glasgow, UK; 4 Edinburgh IBD Unit, Western General Hospital, Edinburgh, UK; 5 Department of Pathology, Western General Hospital, Edinburgh, UK

**Keywords:** Colorectal cancer, Inflammation

## Abstract

Inflammatory Bowel Disease-associated colorectal cancer (IBD-CRC) is a known and serious complication of Inflammatory Bowel Disease (IBD) affecting the colon. However, relatively little is known about the pathogenesis of IBD-associated colorectal cancer in comparison with its sporadic cancer counterpart. Here, we investigated the function of *Dock2*, a gene mutated in ~10% of IBD-associated colorectal cancers that encodes a guanine nucleotide exchange factor (GEF). Using a genetically engineered mouse model of IBD-CRC, we found that whole body loss of *Dock2* increases tumourigenesis via immune dysregulation. *Dock2*-deficient tumours displayed increased levels of IFNγ-associated genes, including the tryptophan metabolising, immune modulatory enzyme, IDO1, when compared to *Dock2*-proficient tumours. This phenotype was driven by increased IFNγ-production in T cell populations, which infiltrated *Dock2*-deficient tumours, promoting IDO1 expression in tumour epithelial cells. We show that IDO1 inhibition delays tumourigenesis in *Dock2* knockout mice, and we confirm that this pathway is conserved across species as IDO1 expression is elevated in human IBD-CRC and in sporadic CRC cases with mutated *DOCK2*. Together, these data demonstrate a previously unidentified tumour suppressive role of DOCK2 that limits IFNγ-induced IDO1 expression and cancer progression, opening potential new avenues for therapeutic intervention.

## Introduction

Inflammatory Bowel Disease–associated colorectal cancer (IBD-CRC) is a long term colonic complication of inflammatory Bowel Disease (IBD) [1]. IBD is a complex, chronic disease affecting the gastrointestinal tract, characterised by immune dysregulation as a result of genetic, environmental and microbiotal factors [2], and is generally classified into two subtypes: Ulcerative Colitis, and Crohn’s Disease. IBD-CRC occurs on a background of colonic inflammation, and occurs in younger patients [3], and is associated with increased mortality compared to sporadic colorectal cancer [3, 4], yet its pathogenesis is poorly understood. There is therefore an urgent need to understand IBD-CRC in greater detail.

The mutational spectrum of IBD-CRC is different from sporadic colorectal cancer with alterations in *TP53* being the most common driver event and *APC* and *KRAS* being mutated at significantly lower rates [5–7]. In addition, alterations in the RHO/RAC signalling pathway are frequently associated with IBD-CRC with exome sequencing studies suggesting a prevalence of 50–70% of tumours having alterations in this pathway [6, 7]. It should be noted however that these data do not imply significant mutation, and the functional effects of these mutations are unknown. RAC proteins have a critical role in several cellular processes including migration, apoptosis, proliferation, and invasion [8–11] but their role in the etiology of IBD-CRC is poorly understood.

Of particular interest are mutations in the dedicator of cytokinesis 2 (*DOCK2*) gene, which have been found in ~10% of IBD-CRC cases [6, 7]. DOCK2 is a 212 kDa protein expressed in both human and mouse, found mainly in peripheral blood cells, and to a lesser extent, the thymus and spleen [12]. DOCK proteins act primarily as guanine nucleotide exchange factors (GEFs) for the RAC/RHO GTPases [13]. GEF activity involves catalysing the release of GDP in exchange for GTP on RAC, resulting in RAC activation [11]. DOCK2 specifically is involved in both the innate and adaptive immune responses and plays a critical role in activation and proliferation of T lymphocytes [14]. DOCK2 was recently identified as a key driver gene in human IBD using network predictive modelling [15]. *Dock2*^*—/—*^ mice are also more sensitive to colitis as a result of *Citrobacter rodentium* infection [16]. As DOCK2 has been linked to both IBD and IBD-CRC in the context of its known physiological effect on immune cells, it is a particularly attractive target to study, and to our knowledge, the functional role of DOCK2 on the development of IBD-CRC has not been previously examined.

Here, we demonstrate loss of *Dock2* in a Dextran Sodium Sulphate (DSS) colitis-induced mouse model of IBD-CRC increases tumour formation. We find this increased tumourigenesis is associated with CD3 + T cell infiltration, increased interferon gamma signalling, elevated expression of the IFNγ target IDO1 and increased tryptophan metabolism in *Dock2*-deficient tumours. In addition, we show elevated production of IFNγ in immune cell populations, including γδ and CD8 T cells, under normal homeostatic conditions suggesting loss of *Dock2* leads to immune cell dysfunction and aberrant IFNγ signalling. IDO1 is elevated in human IBD-CRC patients and pharmacological inhibition of IDO1 activity in *Dock2-*deficient mice abrogates tumourigenesis. Together, these results outline a novel functional role for aberrant IFNγ producing immune dysfunction in promoting IBD-CRC via modulation of IDO1-mediated tryptophan metabolism and suggest potential therapeutic avenues for targeting human disease.

## Results

### Loss of *Dock2* leads to increased tumour formation in vivo

**Fig. 1:**
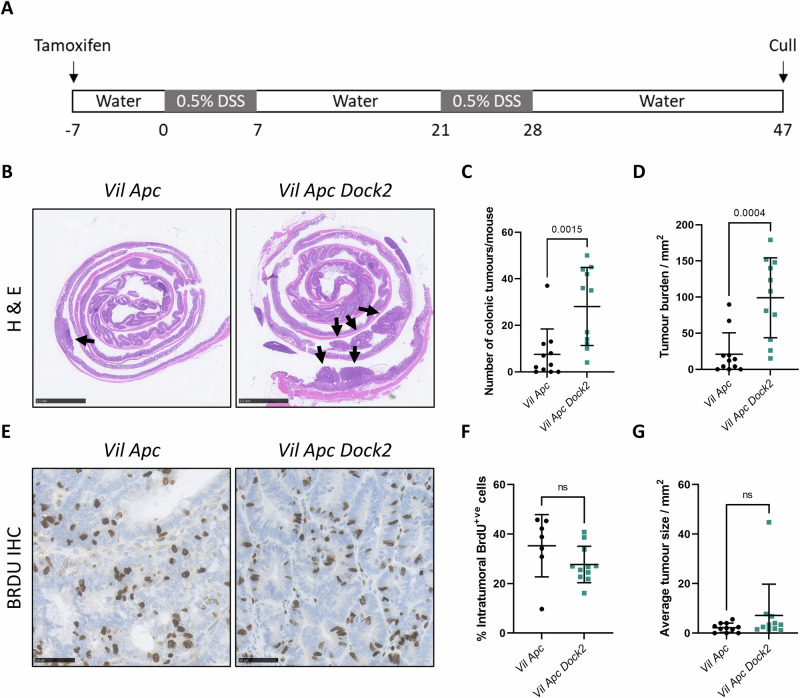
*Dock2* deletion promotes colitis associated colorectal cancer. **A** Schematic detailing the *Apc* loss-mediated mouse model of IBD-associated colorectal cancer, including inducible gene modification with tamoxifen, two rounds of 0.5% DSS, and termination at day 47 post DSS administration. **B** Representative H&E-stained colonic Swiss rolls, detailing tumour burden in *Vil Apc* and *Vil Apc Dock2* mice (black arrows). Scale bars are 2.5 mm. **C** Tumour number in *Vil Apc* and *Vil Apc Dock2* mice. N = 11 vs 11 mice. **D** Total tumour burden in *Vil Apc* and *Vil Apc Dock2* mice. N = 11 vs 11 mice. **E** Representative BrdU-stained *Vil Apc* and *Vil Apc Dock2* tumours. Scale bars are 50 μm. **F** Quantification of BrdU staining. N = 7 vs 11 mice. **G** Average tumour size in *Vil Apc* and *Vil Apc Dock2* mice. N = 11 vs 11 mice. Data represented as mean and error bars SD. All statistical analysis for this figure was performed using two-tailed Mann–Whitney test. Exact p values are indicated in the panels.

To determine whether loss of *Dock2* promotes inflammation-associated CRC development we crossed mice carrying a whole mouse ‘deletion first’ *Dock2*^*tm1a*^ allele to a well characterised *villin-cre*^*ERT2*^ *Apc*^*fl/+*^ intestinal CRC model [17] (Fig. S1A, B) generating cohorts of control *villin-cre*^*ERT2*^ *Apc*^*fl/+*^ (*Vil Apc*) and experimental *villin-cre*^*ERT2*^ *Apc*^*fl/+*^ *Dock2*^*tm1a/tm1a*^ (*Vil Apc Dock2*) mice. Following induction with tamoxifen, mice were subjected to two rounds of colitis-inducing DSS treatment, aged to 47 days where after colons were harvested for histological analysis (Fig. 1A). Loss of *Dock2* expression due to the ‘deletion first’ allele was confirmed in colonic tissue by mRNA and protein analysis and in thymus by RT-qPCR (Fig. S1C–E). *Vil Apc Dock2* mice developed more colonic tumours than controls and had an increased overall tumour burden (Fig. 1B–D). Average tumour size and tumour cell proliferation was not different between groups (Fig. 1E–G) suggesting increased tumourigenesis was not due to accelerated tumour growth (Fig. 1F, G).

### Tumours in mice lacking *Dock2* show an immune phenotype, elevated interferon gamma signalling, and upregulation of IDO1

**Fig. 2:**
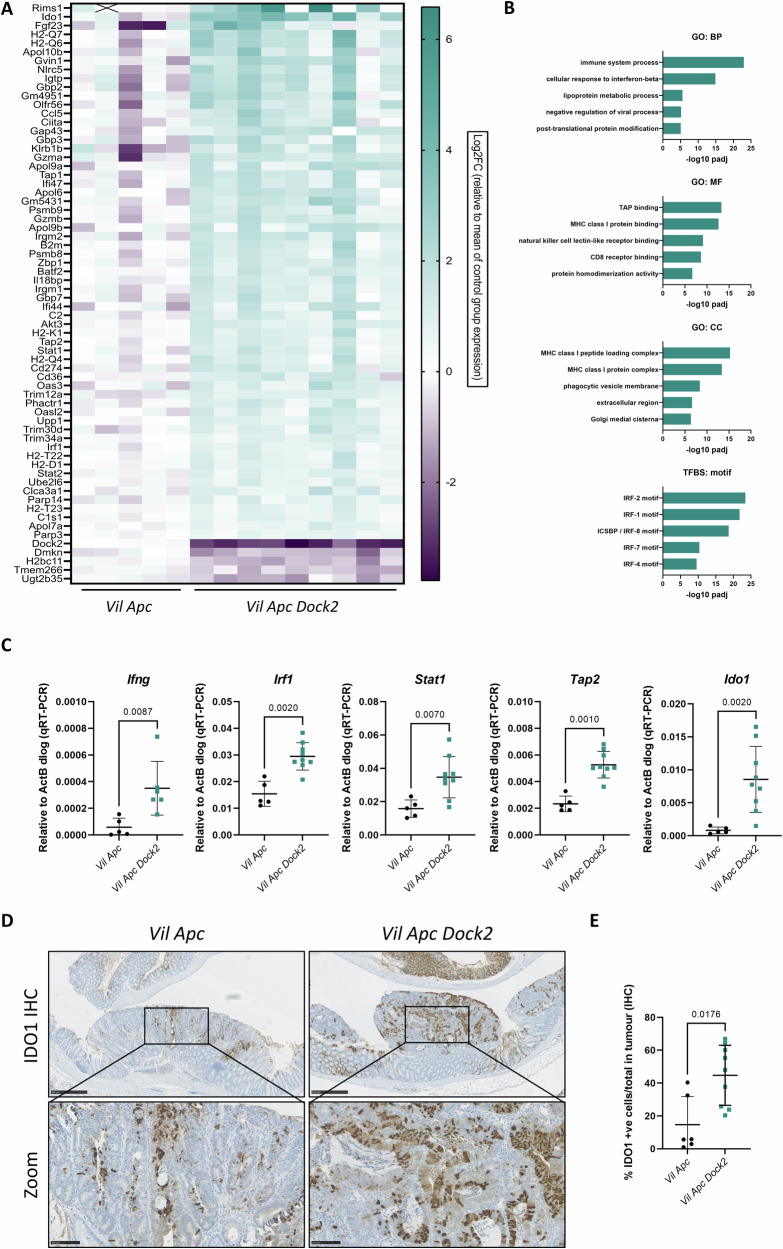
*Dock2* deficient tumours have elevated IFNγ signalling. **A** Heatmap of genes with significantly altered expression in *Vil Apc Dock2* tumour identified by RNAseq. **B** Gene ontology analysis of *Vil Apc* vs *Vil Apc Dock2* tumours. The top 5 non-redundant Biological Processes (BP), Molecular Functions (MF), Cellular Components (CC) and Putative Transcription Factor Binding Sites (TFBS) are listed and corresponding –log10 padj shown. **C** RT-qPCR analysis of IFNγ target gene expression in *Vil Apc* and *Vil Apc Dock2* tumours. N = 5 vs 6 tumours (*Ifng*) or 5 vs 9 tumours (others). **D** Representative IDO1-stained *Vil Apc* and *Vil Apc Dock2* tumours. Scale bars are 500 μm and 100 μm (zoom). **E** Quantification of IDO1 staining. N = 6 vs 9 tumours. Data represented as mean and error bars SD. All statistical analysis for this figure was performed using two-tailed Mann–Whitney test. Exact p values are indicated in the panels.

To identify potential molecular mechanisms mediating increased tumourigenesis following *Dock2* deletion we carried out RNAseq on 5 vs 9 colonic tumours dissected from *Vil Apc* and *Vil Apc Dock2* mice. This analysis revealed 80 transcripts with altered expression (FC > ±1.5, padj < 0.05; 65 upregulated, 15 downregulated) (Fig. 2A and Table S1). *Dock2* was the most downregulated gene, with a logFC of −2.89 and an adjusted p value = 8.87 × 10^−35^, in line with the minimal *Dock2* expression observed in the *Vil Apc Dock2* tumours (Fig. S1C, D). Gene ontology (GO) analysis of our dataset identified enrichment of numerous immune related pathways as being activated in *Dock2* deficient tumours (Fig. 2B and Table S2). These included pathways involved in immune system processes, MHC protein binding and response to interferon signalling. Additionally, transcription factor binding site (TFBS) analysis demonstrated an enrichment of putative targets of interferon regulatory factors (IRFs) (Fig. 2B and Table S2). Furthermore, gene set enrichment analysis (GSEA) identified enrichment of interferon gamma and alpha response hallmark gene sets with genes upregulated in our dataset (Fig. S2A; IFNγ p < 0.001 and IFNα p = 0.005). Together, this suggests loss of *Dock2* leads to dysregulation of immune system processes and, in particular, activation of interferon signalling. Quantitative RT-PCR was used to validate a selection of the identified interferon related genes with all tested genes showing the expected transcriptional changes (Fig. 2C). One of the most upregulated genes following *Dock2* loss was indoleamine 2, 3-dioxygenase 1 (*Ido1*). *Ido1* is a previously described target of IFNγ signalling with known tumour promoting, immunomodulatory functions [18, 19]. Therefore, we chose to investigate IDO1 in more detail. We further validated this upregulation at protein level observing upregulation of IDO1 protein in *Dock2* deficient tumours by IHC and Western blot (Fig. 2D, E and Fig. S2B, C). To define in better detail the cellular compartment expressing IDO1 we co-stained *Vil Apc Dock2* tumour tissue for epithelial and immune cell markers alongside IDO1. IDO1 expression was exclusively co-localised with CDH1 expression and we did not observe co-expression with CD3 or CD8 markers (Fig. S2D, E). Together, this suggests an induction of IFNγ signalling in the tumour epithelium of *Dock2* deficient mice. We also observed increased *Ido1* but not *Ifng* expression in normal tissue adjacent to tumours demonstrating this effect is not restricted to tumour cells (Fig. S2F).

**Fig. 3:**
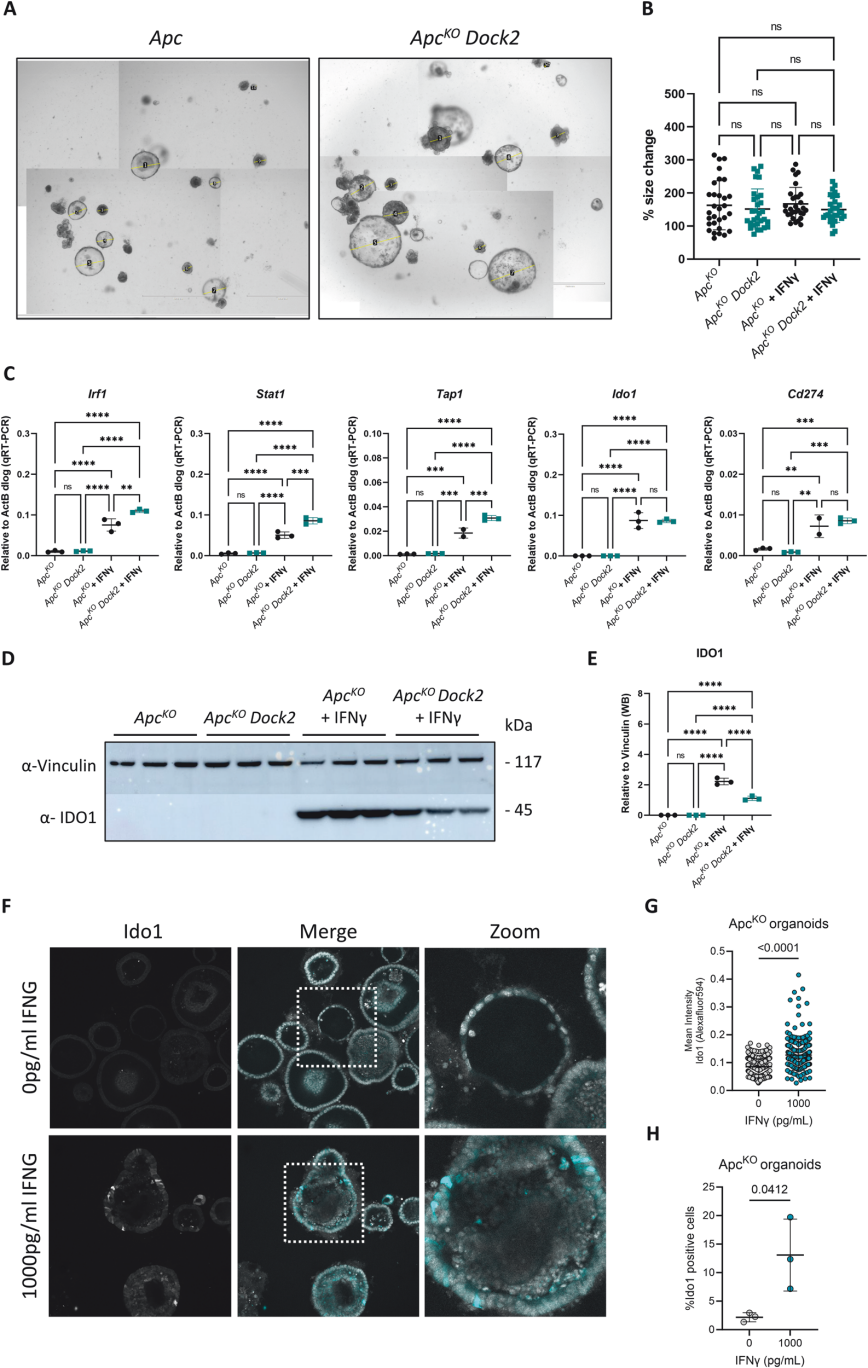
IFNγ signalling is equivalent in normal and *Dock2* deficient tissue. **A** Collage overview of *Apc*^*KO*^ and *Apc*^*KO*^ *Dock2* organoids. **B** Quantification of *Apc*^*KO*^ and *Apc*^*KO*^ *Dock2* organoid size change 24 h post IFNγ treatment. N = 30 vs 30 vs 30 vs 30 organoids. **C** RT-qPCR analysis of IFNγ target gene expression in *Apc*^*KO*^ and *Apc*^*KO*^ *Dock2* organoids 24 h post IFNγ treatment. N = 3 vs 3 vs 3 vs 3 independent technical replicates and 3 vs 3 vs 2 vs 3 independent technical replicates (*Tap1*). **D** Western blot analysis of IDO1 expression in *Apc*^*KO*^ and *Apc*^*KO*^ *Dock2* organoids 24 h post IFNγ treatment. **E** Quantification of IDO1 Western blot. N = 3 vs 3 vs 3 vs 3 independent technical replicates. **F** Immunofluorescence staining of Ido1 expression in *Apc*^*KO*^ organoids 24 h post IFNγ treatment. **G** Quantification of Ido1 staining intensity. N = 572 vs 232 organoid cells. **H** Quantification of % Ido1 positive cells. N = 3 vs 3 independent technical replicates. Data represented as mean and error bars SD. Statistical analysis for (**B**), (**C**) and (**E**) was performed using ordinary one-way ANOVA with Tukey’s multiple comparisons. Statistical analysis for (**G**) and (**H**) was performed using student T-test *p < 0.05, **p < 0.01, ***p < 0.001, ****p < 0.0001.

To further investigate this, we determined whether colonic tumour epithelial cells respond to exogenous IFNγ. Colonic organoids were derived from control and *Dock2*^*tm1a/tm1a*^ (*Dock2*) mice and *Apc* deleted using CRISPR to mimic tumour development in an inflammation naïve setting (Fig. S3A). *Apc*^*KO*^ and *Apc*^*KO*^ *Dock2* organoids were treated with mouse IFNγ for 24 h. There were no changes in organoid growth (Fig. 3A, B) but we observed an upregulation of genes identified in our RNAseq dataset confirming they are IFNγ targets in colonic epithelial cells and indicating a key role for type II interferon signalling in the epithelial response to interferon stimulation (Fig. 3C). This included increased *Ido1* expression which was also observed at protein level (Fig. 3D–H). Interestingly, whilst there was a modest increase in the activation of some IFNγ target genes in IFNγ treated *Apc*^*KO*^ *Dock2* organoids compared to IFNγ treated controls this was not the case for *Ido1* mRNA or protein, the activation of which was broadly the same (Fig. 3C–E). This was in contrast to our in vivo data showing an upregulation of *Ido1* in *Dock2* deficient mice. Thus, the responsiveness of *Dock2* deficient and normal tumour organoids to IFNγ stimulation is broadly similar. Together, this suggests the increased IFNγ response observed in vivo may be due to elevated infiltration of IFNγ producing cells in *Dock2* deficient tumours. To further test this, we analysed the dose dependency of *Apc*^*KO*^ organoids to IFNγ stimulation. We found that IDO1 protein and mRNA expression was induced in a dose dependent manner by increasing concentrations of IFNγ (Fig. S3B–D). Additionally, we found that *Apc*^*KO*^ organoids did not express detectable levels of *Ifng* mRNA, even following inflammatory stimulus with TNFα. Together, this supports the hypothesis that increased IFNγ production, from non-cell autonomous sources, is driving elevated IDO1 expression in tumour epithelial cells in *Dock2* deficient mice. Therefore, we sought to identify potential sources of this IFNγ production.

### Tumours in mice lacking *Dock2* are infiltrated by CD3+ and γδ T lymphocytes

**Fig. 4:**
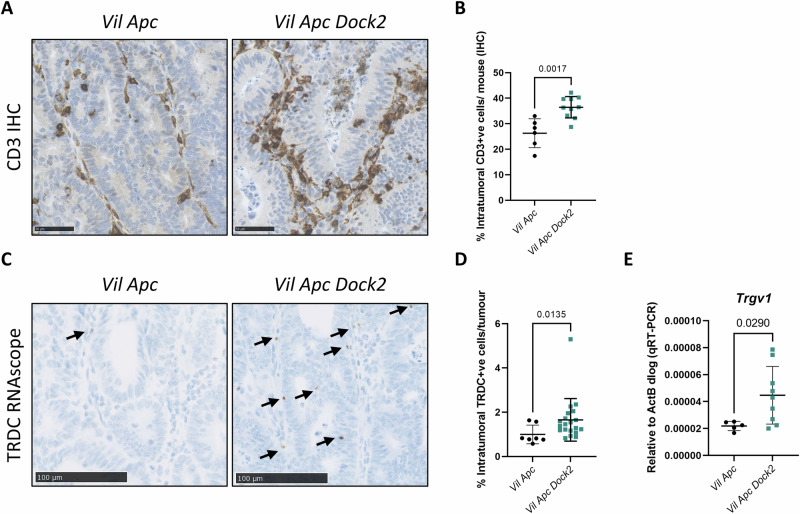
*Dock2* deficient tumours have increased γδ T cell infiltration. **A** Representative CD3-stained *Vil Apc* and *Vil Apc Dock2* tumours. Scale bars are 50 μm (**B**) Quantification of CD3 staining. Values represent % of total tumour cells. N = 6 vs 10 mice. **C** Representative images of RNAscope for TRDC in tumours of *Vil Apc* and *Vil Apc Dock2* mice. Scale bars are 100 μm. **D** Quantification of TRDC staining. Values represent % of total tumour cells. N = 7 vs 20 tumours. **E** RT-qPCR analysis of *Trgv1* expression in *Vil Apc* and *Vil Apc Dock2* tumours. N = 5 vs 9 tumours. Data represented as mean and error bars SD. All statistical analysis for this figure was performed using two-tailed Mann–Whitney test. Exact p values are indicated in the panels.

As DOCK2 primarily functions as an immune modulator we examined whether immune cell dysregulation might be responsible for the observed IFNγ stimulation and thus impacting tumour formation in *Dock2* deleted mice. We first investigated lymphoid and myeloid cell populations using immunohistochemistry and found an increase in intratumoural CD3 + T cell infiltration in *Vil Apc Dock2* tumours (Fig. 4A, B) but not of macrophages or neutrophils (Fig. S4A, B). Further investigations of different T cell subtypes demonstrated that whilst CD4+ and CD8 + T cell infiltration was the same between groups (Fig. S4C, D), there were more γδ T cells in *Vil Apc Dock2* tumours than control tumours (Fig. 4C, D). The expression of *Trgv1*, the gene encoding T cell receptor Vγ1 chain, but not *Trgv4, Trgv5, Trgv6* or *Trgv7* was also upregulated in tumours of *Vil Apc Dock2* mice (Fig. 4E and S4E). Infiltrating Vγ1 γδ T cells have previously been identified as a potential source of IFNγ in tumours suggesting this immune cell population may be partly mediating IFNγ signalling in *Dock2* deficient tumours.

### γδ T cells from mice lacking *Dock2* produce more IFNγ in vivo

**Fig. 5:**
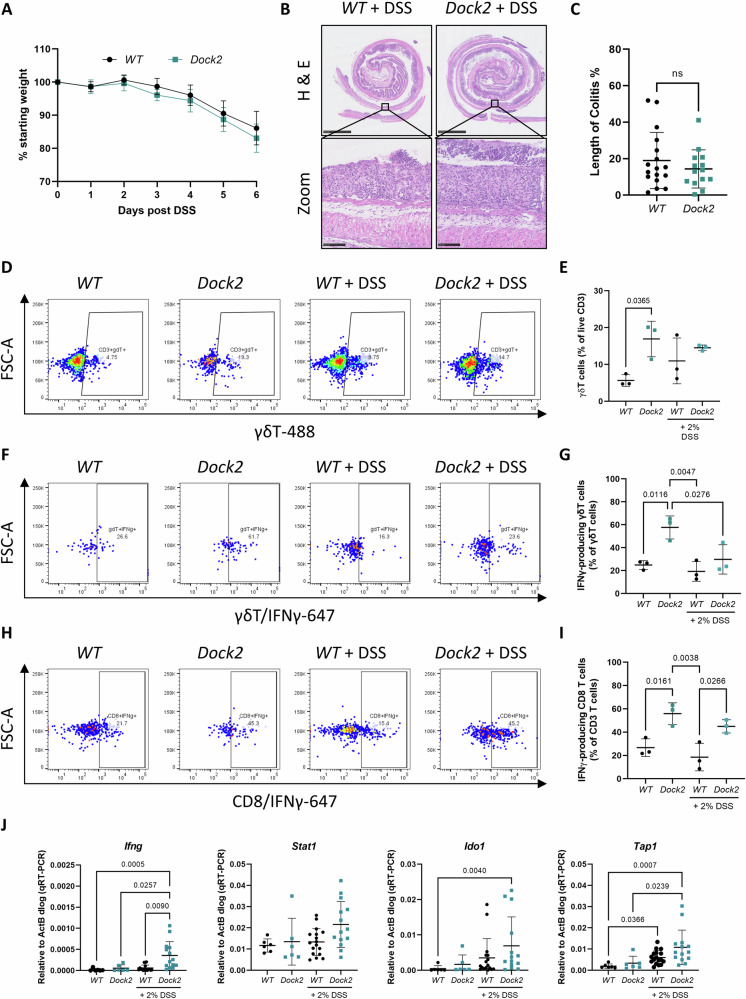
IFNγ producing γδ T cells are increased in *Dock2* deficient colons independently of acute inflammation. **A** Comparative weights of *WT* and *Dock2* mice during treatment with 2% DSS. **B** Representative H&E-stained colonic Swiss rolls, detailing extent of colitis in *WT* and *Dock2* mice. Representative areas of epithelial erosion are highlighted. Scale bars are 2.5 mm and 100 μm (zoom). **C** Quantification of colitic area in *WT* and *Dock2* mice. N = 17 vs 14 mice. **D** Representative plots of CD3+ γδ T cells in *WT* and *Dock2* colons at baseline or following acute DSS treatment. **E** Quantification of CD3+ γδ T cells. N = 3 vs 3 vs 3 vs 3 mice. **F** Representative plots of IFNγ producing γδ T cells in *WT* and *Dock2* colons at baseline or following acute DSS treatment. **G** Quantification of IFNγ producing γδ T cells. N = 3 vs 3 vs 3 vs 3 mice. **H** Representative plots of IFNγ producing CD8 + T cells in *WT* and *Dock2* colons at baseline or following acute DSS treatment. **I** Quantification of IFNγ producing CD8 + T cells. N = 3 vs 3 vs 3 vs 3 mice. **J** RT-qPCR analysis of IFNγ target gene expression in *WT* and *Dock2* colons at baseline or following acute DSS treatment. N = 6 vs 6 vs 17 vs 14 mice. Data represented as mean and error bars SD. Statistical analysis for (**C**) was performed using two-tailed Mann–Whitney test. Statistical analysis for (**E**), (**G**), (**I**) and (**J**) was performed using ordinary one-way ANOVA with Tukey’s multiple comparisons. Exact p values are indicated in the panels.

We next examined in more detail whether γδ T-cells are a source of IFNγ in our *Dock2-*deficient mouse model and whether this phenotype is intrinsic to *Dock2* loss or requires exogenous inflammatory stimulus. To address this, we analysed the effects of *Dock2* deletion on colonic immune cell populations under both homeostatic and inflammatory conditions. Acute colonic damage and inflammation was induced by treatment with 2% DSS. Monitoring of mice during treatment indicated no significant differences in disease severity (as indicated by weight loss) between control and *Dock2* deficient mice (Fig. 5A). After 7 days treatment (5 days DSS + 2 days normal drinking water) mice were culled and colons harvested (Fig. S5A). Histological analysis indicated no significant difference in the extent of mucosal damage or CD3 + T cell infiltration between control and *Dock2* deficient mice (Figs. 5B, C and S5B, C) demonstrating loss of *Dock2* does not increase susceptibility to DSS induced colitis. To investigate the potential for immune cell dysregulation during acute colitis in more detail we carried out flow cytometry analysis of different lymphoid populations (Fig. S5D). Consistent with our previous findings in cancer models, we found γδ T cells were more abundant in *Dock2* deficient than control colons (Fig. 5D, E). Notably, this increase was found under normal, homoeostatic conditions in the absence of DSS induced inflammatory stimulus. Indeed, upon DSS treatment, the proportion of γδ T cells was not different between control and *Dock2* deficient mice (Fig. 5D, E). We next determined whether γδ T cells could act as a source of IFNγ explaining the increased IFNγ activation observed in *Dock2* deficient mice. Flow cytometry analysis showed that a higher proportion of both γδ T cells and CD8 + T cells expressed IFNγ in *Dock2* deficient colons. Again, this increase was present in the absence of DSS treatment, suggesting *Dock2* loss leads to an intrinsic immune cell dysfunction manifested by increased infiltration of IFNγ producing γδ T cells (Fig. 5F–I). To determine whether other immune populations could contribute to increased IFNγ production we further analysed IFNγ levels in CD3 positive and CD3 negative cells by flow cytometry. We found that the majority of IFNγ producing cells were CD3 positive but IFNγ was also produced by CD3 negative cells and the proportion of these cells was also significantly higher in *Dock2* deficient mice (Fig. S5F, G). Together, this suggests *Dock* loss leads to immune dysregulation, characterised by increased IFNγ production in multiple immune subtypes including, but not restricted to, γδ T cells and CD8 + T cells.

To determine whether this led to increased IFNγ target gene expression in the colon we carried out transcriptional analysis of previously identified IFNγ responsive genes. Under homeostatic conditions *Dock2* loss was not sufficient to increase IFNγ signalling in the colon but following DSS treatment a number of IFNγ responsive genes were induced (Fig. 5J). Therefore, *Dock2* deficient colonic epithelium is not inherently IFNγ activated, rather, it responds to inflammation to induce and maintain this state. To investigate more broadly, we also examined expression of γδ T cell markers, and some IFNγ responsive genes in thymus from untreated control and *Dock2* deficient mice. These mice exhibit significantly higher expression of various γδ T cell markers but not IFNγ target genes (Fig. S5H). Together, these data suggest *Dock2* deficient mice have increased infiltration of IFNγ-producing immune cells, but exogenous inflammatory stimuli are needed to induce robust IFNγ activation and IDO1 expression in the colonic epithelium.

### IDO1 expression is increased in human IBD-CRC and IDO1 inhibition abrogates tumourigenesis in *Dock2* deficient mice

**Fig. 6:**
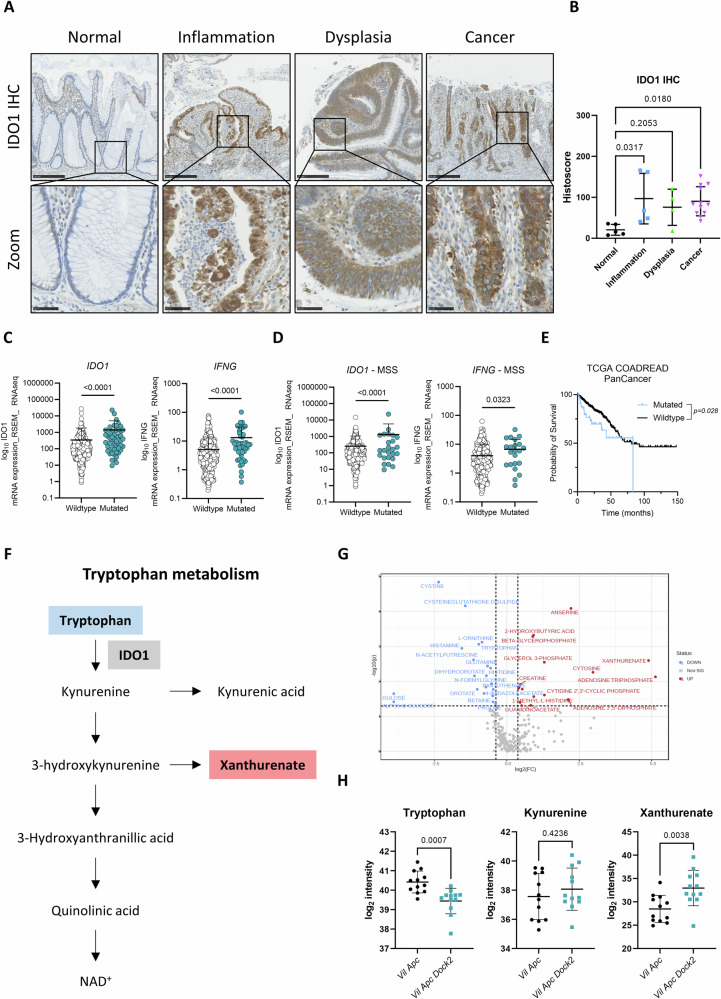
IDO1 is elevated in human IBD-CRC and alters tryptophan metabolism in vivo. **A** Representative IDO1-stained human normal colon, inflamed colon, dysplastic colon and IBD associated cancer (all regions of the same human resection specimen). Scale bars are 250 μm and 50 μm (zoom) (**B**) Quantification of IDO1 staining. N = 5 vs 5 vs 4 vs 12 patient samples. **C** Expression of *IDO1* and *IFNG* in CRC patients with wildtype or mutated *DOCK2*. **D** Expression of *IDO1* and *IFNG* in MSS CRC patients with wildtype or mutated *DOCK2*. **E** Survival plot of TCGA CRC dataset grouped on the mutational status of *DOCK2*. Note reduced survival of *DOCK2* mutant patients. **F** Schematic outlining the tryptophan metabolism pathway. Note IDO1 catalysers the conversion of tryptophan to kynurenine. Metabolites altered in *Vil Apc Dock2* tumours are highlighted in blue if downregulated (tryptophan) or red if upregulated (xanthurenate). **G** Volcano plot showing the polar metabolites filtered using p > 0.05 and FC > 1.3. **H** Individual value plots of log_2_ intensity values of selected metabolites. N = 12 vs 12 tumours. Data represented as mean and error bars SD. Statistical analysis for (**B**) was performed using ordinary one-way ANOVA with Tukey’s multiple comparisons. Statistical analysis for (**C**) and (**D**) was performed using student T-test. Statistical analysis for (**H**) was performed using two-tailed Mann–Whitney test. Exact p values are indicated in the panels.

To determine whether the observations from our model are recapitulated in human disease we investigated the expression of the IFNγ target IDO1 in a set of human samples encompassing different stages of IBD-CRC disease progression. IHC analysis showed that IDO1 expression is significantly increased in inflamed tissue compared to normal colon and this increased expression persists through to the development of cancer (Fig. 6A, B).

To extend these analyses we utilised the TCGA dataset of sporadic CRC. *DOCK2* is mutated in ~10% of cases and in samples where *DOCK2* is mutated, expression of both *IFNG* and *IDO1* was significantly higher than non-mutated samples (Fig. 5C). Importantly, this is also observed in microsatellite stable (MSS) CRC which is generally immune suppressed (Fig. 5D). We also found that *DOCK2* mutation and/or low *DOCK2* expression correlates poor prognosis in these patients, further supporting a tumour suppressor role for this protein (Figs. 6E and S6A, B).

IDO1 is a tryptophan catabolic enzyme that catalysers the conversion of tryptophan to kynurenine and other downstream metabolites (Fig. 6F). This pathway plays an important immunomodulatory role by regulating the activity of both regulatory and effector T cells [20–22]. We next investigated whether the increased IDO1 observed in *Dock2* deficient tumours impacted on tryptophan metabolism. To identify metabolic differences between *Vil Apc* and *Vil Apc Dock2* tumours, we performed liquid chromatography mass spectrometry (LC-MS). We extracted metabolites using a solid-liquid biphasic extraction [23] from 12 tumours of each group and analysed the aqueous fraction by high-resolution HILIC LC-MS [24]. We identified numerous changes in metabolite levels in *Dock2* deficient tumours (Figs. 6G, S6C, S6D and Table S3). Consistent with the elevated expression of IDO1, we observed a depletion of tryptophan following *Dock2* deletion (Fig. 6G, H). Interestingly, we did not observe differences in the levels of kynurenine but the downstream metabolite xanthurenate was elevated in *Vil Apc Dock2* tumours suggesting a rapid conversion of kynurenine via this pathway (Fig. 6F–H).

**Fig. 7:**
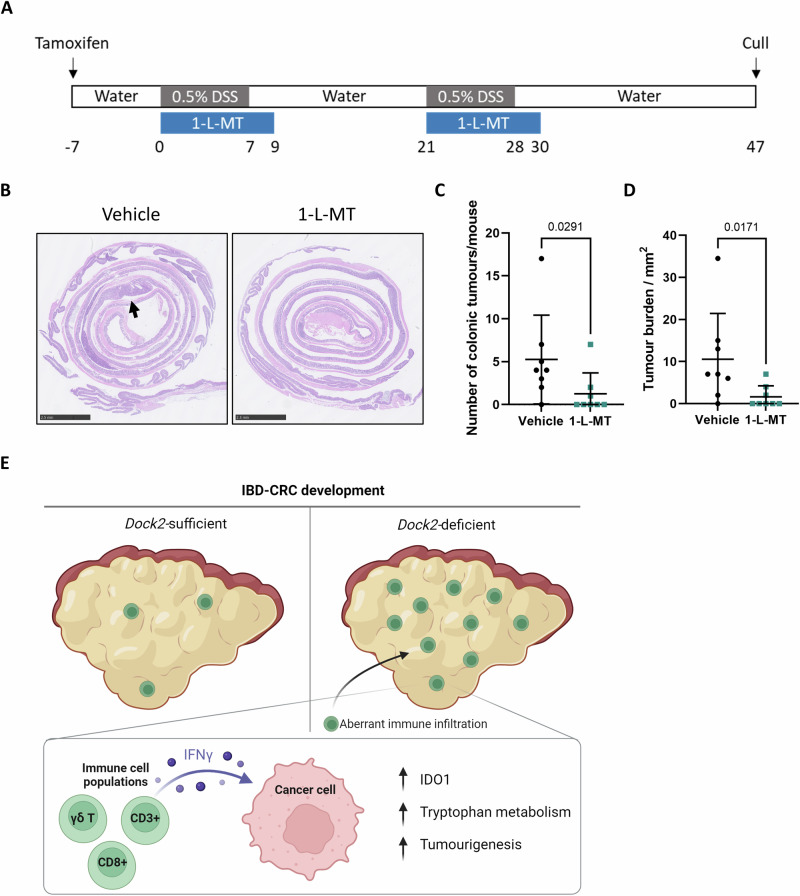
IDO1 inhibition abrogates tumourigenesis in *Vil Apc Dock2* mice. **A** Schematic outlining IDO inhibitor (1-L-MT) treatment regimen in *Vil Apc Dock2* mice. **B** Representative H&E-stained colonic swiss rolls, detailing tumour burden in vehicle and IDO1 inhibitor (1-L-MT) treated *Vil Apc Dock2* mice (black arrows). Scale bars are 2.5 mm. **C** Tumour number in vehicle and IDO1 inhibitor treated *Vil Apc Dock2* mice. **D** Total tumour burden in vehicle and IDO1 inhibitor treated *Vil Apc Dock2* mice. N = 8 vs 8 mice. **E** A model outlining the proposed role of *Dock2* in suppressing inflammation induced colorectal cancer. Data represented as mean and error bars SD. All statistical analysis for this figure was performed using two-tailed Mann–Whitney test. Exact p values are indicated in the panels.

To further test the functional significance of elevated IDO1 expression and tryptophan metabolism in *Dock2* deficient tumours we next examined the effect of IDO1 inhibition on tumourigenesis. We treated *Vil Apc Dock2* mice with the IDO1 inhibitor 1-L-MT whilst undergoing repeated rounds of DSS induced colitis (Fig. 7A). Mice were culled at 47 days post first day of DSS and tissue harvested for analysis. Consistent with a functional role for IDO1 expression in driving IBD-CRC development, mice treated with 1-L-MT had a significantly lower tumour number and tumour burden compared to mice treated with vehicle (Figs. 7B–D and S7A). Importantly, this was not due to IDO1 inhibition impacting on severity of colitis, confirming the tumour promoting effects of increased epithelial IDO1 activity following Dock2 deletion (Fig. S7B). Together, our data implicate *Dock2* loss of function in the development of IBD-CRC. Mechanistically, this is via immune dysregulation leading to increased infiltration of IFNγ producing T cells, driving IDO1 expression.

## Discussion

Taken together, these results suggest that loss of *Dock2* leads to immune dysregulation, enhancing tumourigenesis via interferon gamma induced expression of IDO1. In mice lacking *Dock2*, IFNγ production is increased in multiple T cell populations, including CD8+ and γδ T cells, and administrating IFNγ to tumour organoids induces robust IDO1 expression suggesting this response is driven by immune cell IFNγ production. Finally, treatment with 1-L-MT, an IDO1 inhibitor, abrogates the increased tumourigenesis observed in mice without *Dock2*.

IDO1 is an enzyme catalysing the conversion of tryptophan to kynurenine [25] and has been directly associated with tumourigenesis, as IDO1+ Paneth cells promote immune evasion in sporadic colorectal cancer [26]. In colitis-associated cancer, conditional loss of IDO1 in the colonic epithelium with AOM/DSS resulted in fewer colonic tumours [18]. Additionally, 1-L-MT, an inhibitor of IDO1, decreases proliferation of colon cancer cell lines [27], as well as reduces tumour burden in AOM/DSS mice [27]. IDO1^−/−^ mice treated with AOM/DSS have separately been shown to develop smaller tumours [28]. We also observed decreased tumourigenesis in a colitis induced *Dock2* deficient tumour model following 1-L-MT treatment, outlining the importance of tryptophan metabolism in modulating colitis-induced CRC. IDO1 is associated with a reduction in immune system activity [25] through several mechanisms. First, macrophages [22] and dendritic cells [21] expressing IDO1 suppress T cell proliferation via decreasing the tryptophan pool. Second, dendritic cells expressing IDO1 expand the regulatory T cell (Treg) population thus modulating inflammatory activity [20]. IDO1 also mediates increased tumourigenesis independently of its effect on T cells, as tumourigenesis was also impaired in IDO1 knock out, immune deficient *Rag1*-knockout mice [29]. Although IDO1 is not abundantly expressed in the colonic epithelium at baseline [19, 30], it is expressed in the epithelium during active IBD [19] consistent with the findings of our study. Interestingly, when overexpressed in the epithelium it results in increased secretory cell differentiation and larger mucus layer in the ileum, as well as reduced sensitivity to DSS [31]. Along these lines, induction of IDO1 affects colitis severity [32]. However, the literature varies with respect to IDO1 loss or inhibition, with both positive and negative results reported in colitis: IDO1-deficient mice are less sensitive to DSS colitis [33], yet mice treated with both IDO1 stimulation and IDO1 inhibitor combined, lose the protective effect of IDO1 stimulation [32]. Thus, activation of IDO1 activity has the potential to mediate colonic homeostasis and tumourigenesis via multiple mechanisms.

Our data strongly implicate IFNγ signalling in driving expression of IDO1, indeed we observed a robust, dose dependent, transcriptional activation of IDO1 following treatment of colonic organoids with recombinant IFNγ. Subsequent analyses identified increased infiltration of CD3 + , CD8+ and γδ T cells as a source of IFNγ in *Dock2* deficient tissue although we cannot rule out other immune populations also playing a role. It is not clear why more IFNγ producing T cells accumulate in tumours formed in *Dock2* mutant mice, a gene that is expressed primarily in hematopoietic cells. These cells may be recruited to tumours by increased expression of chemokines or they may proliferate in situ. Neither is it clear why a predominance of IFNγ-producing T cells, normally associated with anti-tumour activity [34–36], correlate with increased tumourigenesis. For example, Vγ1 γδ T cells play a role in counteracting tumour cell survival through communication with intraepithelial lymphocytes expressing the Vγ7 TCR [37]. Additionally, IFNγ producing CD8 + T cells are known mediators of anti-tumour immunity [38]. On the other hand, IFNγ can contribute to immune cell evasion, in particular via modulation of IDO1 expression [38]. Given that T cells lack *Dock2* expression in the model used here, it is tempting to speculate that the absence of DOCK2 suppresses their cancer-killing functions via preventing synapse formation with target cancer cells, a previously described function of DOCK2 [39]. This defective recognition of cancer cells together with the upregulation of IFNγ in DOCK-2 deficient T cells may result in tumour promotion via increased expression of IDO1. Anti-tumourigenic effects of IDO1, such as induction of T cell apoptosis [40], were not observed either, arguing against immune escape by T cell ablation. It is reasonable, therefore, to suggest that these increased *Dock2* deficient IFNγ producing T cells are dysfunctional. Thus, despite accumulating in the colon they are not sufficient to induce an anti-tumour microenvironment, rather via induction of IDO1 expression, promote one that supports tumour development.

Despite these open questions it appears clear that *Dock2* deficiency leads to increased tumourigenesis in an IDO1 dependent manner. Additionally, we found increased levels of IDO1 in IBD-CRC samples and in patients with sporadic CRC carrying mutations in the *DOCK2* gene. Furthermore, *DOCK2* mutational status was correlated with poor survival outcomes in sporadic CRC. Our identification of this pathway, and its relevance in human disease, could therefore help inform the clinical management of IBD-CRC. With regards to this, it is important first to note the status of IDO1 inhibitors in recent clinical trials. Several trials across multiple solid tumour types have revealed disappointing results [41]. However, these trials have not selected patients based on IDO1 tumour positivity, and none of these trials have occurred in patients with colorectal cancer, particularly following inflammation. Therefore, it remains to be seen whether IDO1 inhibition has a role in the treatment of inflammation-associated colorectal cancer, and this potential needs to be fully explored. Alternatively, this poor performance may indicate that IDO1 activity is not required to fuel tumour growth in established cancers. In this case, IDO1 inhibitors may be more efficacious when used as chemopreventative agents, preventing the initiation of tumourigenesis. It is worth noting that in our model, we inhibited IDO1 during DSS treatment, suggesting a key role during colonic damage and regeneration cycles. Therefore, IDO1 inhibitors may be particularly beneficial to patients with IBD and at high risk of developing cancer, in particular those carrying previously identified high risk *DOCK2* mutations [15]. Additionally, IDO1 activity, identified through increased xanthurenate, has been shown to negatively correlate with intestinal inflammation itself, and modification upstream of xanthurenate has protected against murine colitis [41]. Therefore, there are several opportunities for biomarker identification, as well as potential therapeutic agents, in both inflammation and inflammation-associated cancer.

To summarise, we have identified an IDO1 induced tryptophan metabolic pathway regulated by IFNγ producing immune cells driving IBD-CRC, highlighting the intricate role of the immune and interferon gamma response in IBD-CRC (Fig. 7E). This enhances our understanding of the etiology of IBD-CRC and identifies these processes as potential therapeutic targets for this complex and chronic disease.

## Materials and methods

### Animal models

#### Mus musculus

All animal experiments were performed in accordance with a UK Home Office license (Project License 70/8885), and were subject to review by the animal welfare and ethics board of the University of Edinburgh. Mice of both genders were used at an age of 6–12 weeks. Mice were bred at the animal facilities of the University of Edinburgh and were kept in 12 h light–dark cycles and were given access to water and food ad libitum. Mice were maintained in a temperature- (20–26 °C) and humidity- (30–70%) controlled environment. Colonies had a mixed background (50% C57Bl6J, 50% S129). The genetic alleles used for this study were as follows: *villin-cre*^*ERT2*^ [42], *Apc*^*fl*^ [43] and *Dock2*^*tm1a*^ (EUCOMM). The *Dock2*^*tm1a*^ allele is generated by insertion of a lacZ and neo containing construct into *Dock2* gene. Insertion of this construct disrupts gene expression, resulting in a ‘knockout-first’ allele. Therefore, the *Dock2*^*tm1a*^ allele is Dock2 knockout in all mouse tissues, including immune cell populations. Mice were genotyped by Transnetyx (Cordoba, USA). At experiment endpoints, mice were humanely sacrificed by cervical dislocation (CD) in line with UK Home Office regulations.

### Animal experiments

Gene deletion in *villin-cre*^*ERT2*^ *Apc*^*fl*^ and *villin-cre*^*ERT2*^ *Apc*^*fl*^ *Dock2*^*tm1a*^ mice was induced as previously described using intraperitoneal tamoxifen at 80 mg/kg [44]. Chemical colitis in experimental animals was triggered using DSS (36,000–50,000 Da, MP Biomedicals), reconstituted in distilled water. Mice were treated with two 7-day cycles of 0.5% DSS, with recovery in between, and aged. Labelling of actively replicating cells was achieved through IP injection of 200 μl of BrdU (Amersham Bioscience), 1–2 h prior to Schedule 1 culling. For the inhibitor experiment, mice were treated with 400 mg/kg 1-LMT, an IDO1 inhibitor, daily for the period of DSS administrations, plus two further days via gavage, or vehicle (0.5% methylcellulose/0.5% Tween 80) without drug. Power calculations based on previous results from similar experiments were carried out prior to the experiment to ensure appropriate sample sizes to determine statistically significant effects. Investigators were blinded to mouse genotype during experiment and data analysis.

### Patient samples

Anonymised archival paraffin embedded human colonic resection specimens from patients with IBD-associated colorectal cancer held within the Lothian NRS Bioresource were obtained following approval (Sample Requests SR1165 and SR1165- AM01 301120; Ethical Approvals 15/ES/0094 and 20/ES/0061). Informed consent was obtained from all subjects. Relevant areas of normal tissue, inflammation, dysplasia and cancer were identified by a pathologist and samples were used for subsequent immunohistochemical analysis.

### Immunohistochemistry

After dewaxing, and where necessary, methacarn-fixed slides were treated with 4% PFA for 10 min. Antigen retrieval at 99 °C was in one of two buffers according to protocol: citrate (pH 6) or EDTA. Hydrogen peroxide was used to prevent endogenous staining at either 1.5% or 3%. Sections then were blocked in 5% goat serum (Sigma) for an hour, before application of the relevant primary antibody at dilutions in 5% goat serum, overnight at 4 °C. The following day the secondary antibody (DAKO Envision) was applied according to manufacturer’s recommendations. Staining for all sections was developed using diaminobenzidine (Thermo Scientific) for three minutes, before counterstaining and mounting. BrdU (BD Transduction (347580)) staining was at 1:500, using a citrate buffer for 25 min heating/30 min cooling, and 10 min hydrogen peroxide 1.5%. CD3 (DAKO (A045229)) was at 1:500, using a EDTA buffer for 15 min heating/30 min cooling, and 10 min hydrogen peroxide 3%. IDO1 (Cell Signalling (51851S)) staining was at 1:100, using a citrate buffer for 20 min heating and 20 min cooling, and 10 min hydrogen peroxide 3%.

### RT-qPCR

Primers used for the amplification of each selected gene are detailed in Table S4. The reaction used SYBR Select Master Mix (Applied Biosystems), together with forward and reverse primer (combined and diluted 1:10), and water. Gene expression levels were examined in duplicate and normalised to *Actb* using 2^^-(ΔΔ Cycle threshold)^ calculations. The protocol involved incubation at 95 °C for 15 min, and then 45 cycles of [denaturation at 95 °C for 10 s, annealing at 60 °C for 30 s, and extension at 72 °C for 30 s]. Primers used for analysis are listed in Table S4.

### RNASeq

RNASeq on individual, isolated tumours was carried out by the Welcome Trust Clinical Research Facility (WTCRF) at the Western General Hospital, Edinburgh. After RNA extraction, samples were checked for RNA quality using a Bioanalyser - all had a RIN of 9.1 or above. Libraries were prepared using 500 ng of each sample with the NEBNEXT Ultra II Directional RNA Library Prep kit (NEB #E7760) and the Poly-A mRNA magnetic isolation module (NEB #E7490). Sequencing was performed using the NextSeq 500/550 High-Output v2.5 Kit (#20024907) on the NextSeq 550 platform (Illumina Inc, #SY-415-1002). All libraries generated greater than 24 million paired end reads. FASTQ files (four lanes per sample) were subsequently uploaded for analysis using DESeq2. Significant genes were considered for pathway analyses using G:profiler (https://biit.cs.ut.ee/gprofiler/). Genes were pre-ranked and analysed for Gene Set Enrichment Analysis (GSEA, v3.0) to generate enrichment plots for Hallmark datasets.

### RNAScope

In-situ hybridisation (ISH) staining was performed on 4 µm formalin fixed paraffin embedded sections (FFPE) which had previously been ovened at 60 °C for 2 h. ISH detection for, mRNA probe for Trdc (449358; Advanced Cell Diagnostics) was performed using RNAScope 2.5 LSx (Brown) detection kit (322700; Advanced Cell Diagnostics) on a Leica Bond Rx autostainer strictly according to the manufacturer’s instructions. To complete ISH staining sections were rinsed in tap water, dehydrated through graded ethanols and placed in xylene. The stained sections were coverslipped in xylene using DPX mountant (CellPath, UK).

### Western blotting

Protein samples were first denatured in NuPAGE LDS sample loading buffer (ThermoFisher Scientific) together with NuPAGE sample reducing reagent at 99 °C for 5 min, before loading on to a gel. Gels used were SDS (NuPAGE) pre-prepared and were either 4–12% Bis-Tris or 3–8% Tris-Acetate, depending on the size of the protein of interest. Once running was complete, proteins were transferred onto a nylon membrane (PVDF, Amersham) in Nupage transfer buffer containing 20% methanol in ddH_2_0. Membranes were then blocked in 5% milk before applying antibody (IDO1 - Cell Signalling (51851S), or Vinculin - Abcam (ab73412) at 1:1000 overnight at 4 °C. Samples were then washed, before applying secondary antibody at a concentration of 1:5000 for an hour at room temperature, before developing using Pierce ECL (ThermoFisher) kits.

### Flow cytometry

1 cm of colon was freshly dissected, 2 cm from the rectum, and digested in a cocktail of 2.5 ml RPMI media (Gibco) containing 5% FCS (in-house) 0.5 mg/ml collagenase (Sigma), 0.5 mg/ml DispaseII (Merck) and 3 mg/ml DNAseI (Roche) in a rocking incubator at 37 °C for 45 min before passing through a 70 μm strainer. 5 × 10^5^ cells were plated per sample, with the remainder being used for unstained control. 150 μl of T cell stimulation media (IMDM, Sigma, plus β-mercaptoethanol 50 μM plus Pen-strep (in-house) +T cell stimulation cocktail (1:500, Invitrogen) was added to each well before placing the plate in the 37 °C incubator for three hours. Each stained well was then resuspended in 50 μl blocking buffer (50 μl FACS buffer and 1 μl fc block (TruStain FcX, Biolegend) for 20 min at 4 °C. Meanwhile an antibody mix was prepared using 1 μl per sample of the relevant extracellular antibody diluted 1:50 in Brilliant Stain buffer (BD Biosciences). 50 μl of antibody mix was added to each sample and the plate was further left at 4 °C for 30 min. 100 μl of Zombie live/dead agent (Biolegend) was added at 1:100 in cold PBS and again incubated for 20 min at 4 °C. Samples were resuspended in 75 μl fixation buffer (Invitrogen), again for 20 min at 4 °C. After incubation, 75 μl 1x permeabilisation buffer (Invitrogen, diluted in distilled water) was added before finally resuspending in FACS buffer and keeping at 4 °C overnight.

The following day 100 μl of intracellular antibody mix (diluted 1:100 in permeabilisation buffer) was added to each sample and the plate was incubated at 4 °C for 30 min. After intracellular antibody incubation, 50 μl permeabilisation buffer was added to each well, before resuspending each well in 100 μl FACS buffer. Samples were then flowed on BD Fortessa™. Antibodies used were Zombie Live/Dead (Biolegend (423107) – UV), CD3 (Biolegend (100219) – PE/Cy7), TCRγδ (Biolegend (118127) – AF 488) and IFNγ (Biolegend (505816) – AF647).

### Organoid generation and culture

Media for organoid culture used Advanced DMEM/F12 (ADF - Gibco) with 5 mls HEPES (Invitrogen), 5 ml glutamine (in-house) and 5 ml Penstrep (in-house) added. Colonic Wild-type and Dock2tm1a/tm1a organoids were generated directly from epithelial tissue culture as part of this project. Colon was harvested and 25 mM EDTA was added for thirty minutes to catch free magnesium, loosening up the crypts from the surrounding tissue. Once the EDTA reaction was terminated the post-washing supernatant was collected and centrifuged and the end pellet was resuspended in ADF and passed through a 70 μm cell strainer before resuspending in BME and plating. Complete media containing 15% R-spondin-conditioned media (in-house – HEK-293 cells stably expressing HA-R-Spondin), 50 ng/ml EGF, 1X B27 (Life Technologies), 1X N2 (Life Technologies), 1% Noggin-conditioned media (in-house - HEK 293 cells stably expressing Noggin), 50% Wnt3a condition media (in-house –L-cells stably transfected expressing Wnt3a) and 3 μM StemMACS (CHIR99021, Abcam) was used in culture. Established organoids were then passaged a week later. Organoids were treated with mouse interferon-γ (Life Technologies), diluted in media at 1 ng/ml. Protein and RNA were collected form organoids at 24 h after administration of IFNγ, to compare to organoids cultured only in normal media.

### Organoid CRISPR

Colonic wild-type and Dock2^tm1a/tm1a^ organoids were further modified to CRISPR *Apc*, rendering them Apc^−/−^ and Apc^−/−^Dock2^tm1a/tm1a^. On day 0 cells were split and plated in complete media. On day three they were again split and plated, with the StemMACS concentration raised, now being at 0.6 μl/ml. Additional factors added were Jagged (1 μM, Eurogentec), ROCK inhibitor (10 μM Y-27632, Tocris), and 1 mM VPA (Sigma). On day five viral infection took place, adding 1 ml Accutase™ (Life Technologies) and 4 μl Y-27632 to the harvested pellet. This reaction took place in a water bath at 37 °C for 3 min before termination of the enzymatic reaction with 1 ml 1% BSA and washing in ADF. 5 × 10^5^ cells were plated per well of a six well plate with virus at an MOI of 50. Two guide RNAs for *Apc*, “APC1” (GGATCTGTATCCAGCCGTTC) and “APC3” (AGATCCTTCCCGACTTCCGT), were used. Media for transduction contained EGF, Noggin, R-spondin and Wnt as above, StemMACS 0.6 μl/ml, 1 μM Jagged, 10 μM Y-27632, 1 mM VPA (1:10000), and polybrene (8 μg/ml, Sigma). 24 h later, the infection media was removed and BME overlaid, with fresh media used (containing all constituents of the transduction media bar the polybrene). Media was changed again 24 h after that, this time replacing with ENRW with StemMACS at 1:200 and Y-27632 10 μM only. Media changes were then carried out as necessary until day 11 when growing organoids were split and R-spondin, Wnt and StemMACS removed.

### Metabolomics

Polar metabolites were extracted using biphasic extraction. On a 1.5 mL Eppendorf vial, 100 µL of methanol containing internal standard (L-Glutamine_13C5,15N2) was added to the tissue samples, sonicated on ice cold bath for 10 min, following by the addition of 300 uL of MTBE. Vials were shacked for 20 min at 8 °C and 100 µL H2O was added to induce phase separation, followed by vertexing it for one minute and centrifuged at max speed for 10 min at 4 °C. The upper phase containing lipids and the lower phase containing the polar metabolites were individually transferred for new vials. The polar phase was placed at −80 °C for one hour to guarantee protein precipitation, centrifuged at max speed for 10 min at 4 °C and transfer to a 96 well plate for HPLC analysis. Samples were analysed on a Dionex UltiMate 3000 LC System (Thermo Scientific, Waltham, Massachusetts, EUA) coupled to a Q Exactive Orbitrap Mass Spectrometer (Thermo Scientific, Waltham, Massachusetts, EUA) operating in polarity switch mode. Chromatographic separation was achieved using a ZIC®-pHILIC 150 × 2.1 mm column (Merck MilliporeSigma, Burlington, Massachusetts, EUA) using a gradient starting from 20% buffer A (20 mM ammonium carbonate 0.1% ammonium hydroxide solution 25%), and 80% B (acetonitrile) to 80% buffer A, 20% buffer B at 18 min and reconditioning the column to the initial condition until 27.5 min. Mass spectrometry data were processed using Skyline [45] on a targeted fashion by matching accurate mass and retention time using a in house library acquired from authentic standards. Statistical analysis was performed using metaboanalyst 5.0 [46].

### Diagrams

Diagrams were created using Biorender under a Premium Plan (K.B.M).

### Quantification and statistical analysis

Statistical analyses were performed using GraphPad Prism software (v8.3 GraphPad Software, La Jolla, CA, USA) performing the tests as indicated in the figure legends or main text. Significance levels were calculated according: p < 0.05 (*), p < 0.01(**) and p < 0.001 (***). P values are reported on graphs where comparisons are statistically significant. On graphs with multiple comparisons, for clarity, non-significant changes are not shown.

### Contact for reagent and resource sharing

Requests for further information, reagents and resources should be directed and will be fulfilled by the Lead Contact, Kevin B Myant: (Kevin.myant@ed.ac.uk).

## Supplementary information

Supplemental figures and legends

Table S1

Table S2

Table S3

Table S4

## Data Availability

All relevant data is available from the authors upon request.
