# Supplementary material for: Loss of DOCK2 potentiates Inflammatory Bowel Disease–associated colorectal cancer via immune dysfunction and IFNγ induction of IDO1 expression
Source: Oncogene. 2024 Sep 7;43(42):3094–107. doi: 10.1038/s41388-024-03135-9 (PMC11473400; doi:10.1038/s41388-024-03135-9)
Supplement: Supplementary file 1 — Supplemental figures and legends [file 41388_2024_3135_MOESM1_ESM.docx]

**Supplemental figures and legends**

Supplemental figures 1 – 7

Supplemental figure legends

Supplemental table legends

**
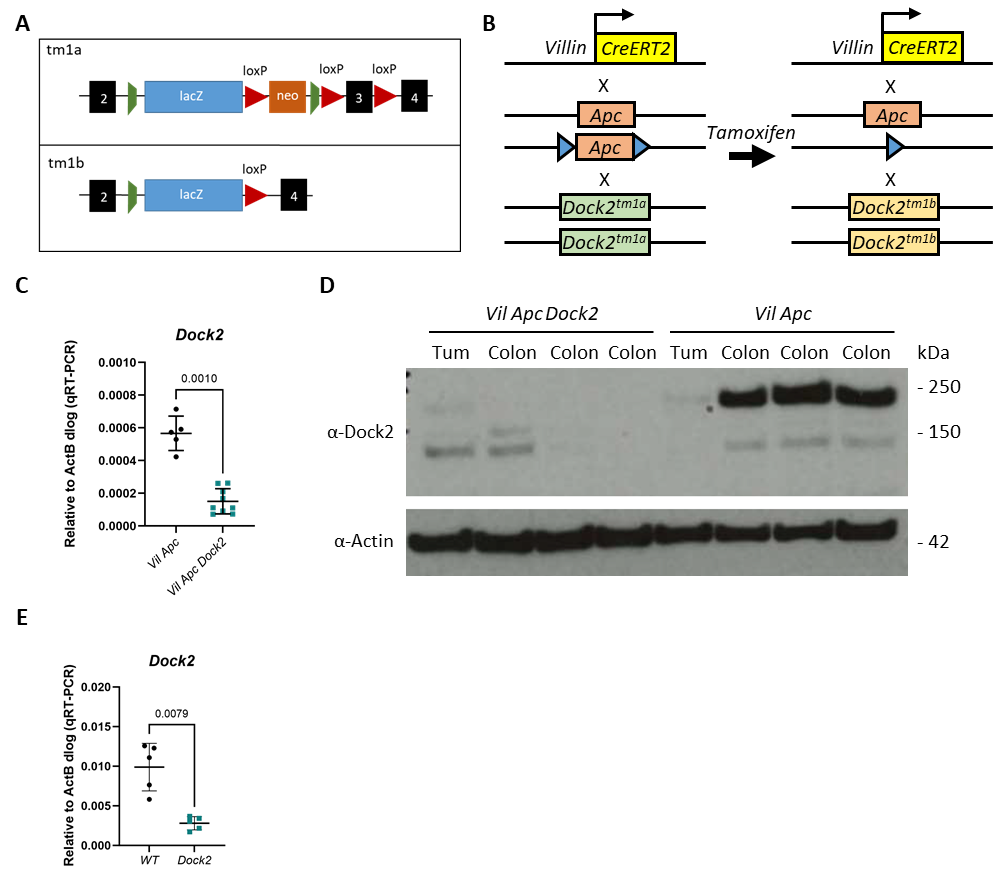
**

**Figure S1. *Dock2* deletion promotes colitis associated colorectal cancer.** (A) Schematic detailing the ‘deletion first’ *Dock2^tm1a^* allele. (B) Schematic outlining the generation and induction of experimental *Vil Apc Dock2* mice. (C) RT-qPCR analysis of *Dock2* expression in *Vil Apc* and *Vil Apc Dock2* tumours. N = 5 vs 9 tumours. (D) Western blot analysis of DOCK2 expression in *Vil Apc* and *Vil Apc Dock2* tumours. (E) RT-qPCR analysis of *Dock2* expression in thymus from *WT* and *Dock2* mice. N = 5 vs 5 mice. Data represented as mean and error bars SD. All statistical analysis for this figure was performed using two-tailed Mann-Whitney test. Exact p values are indicated in the panels.

**
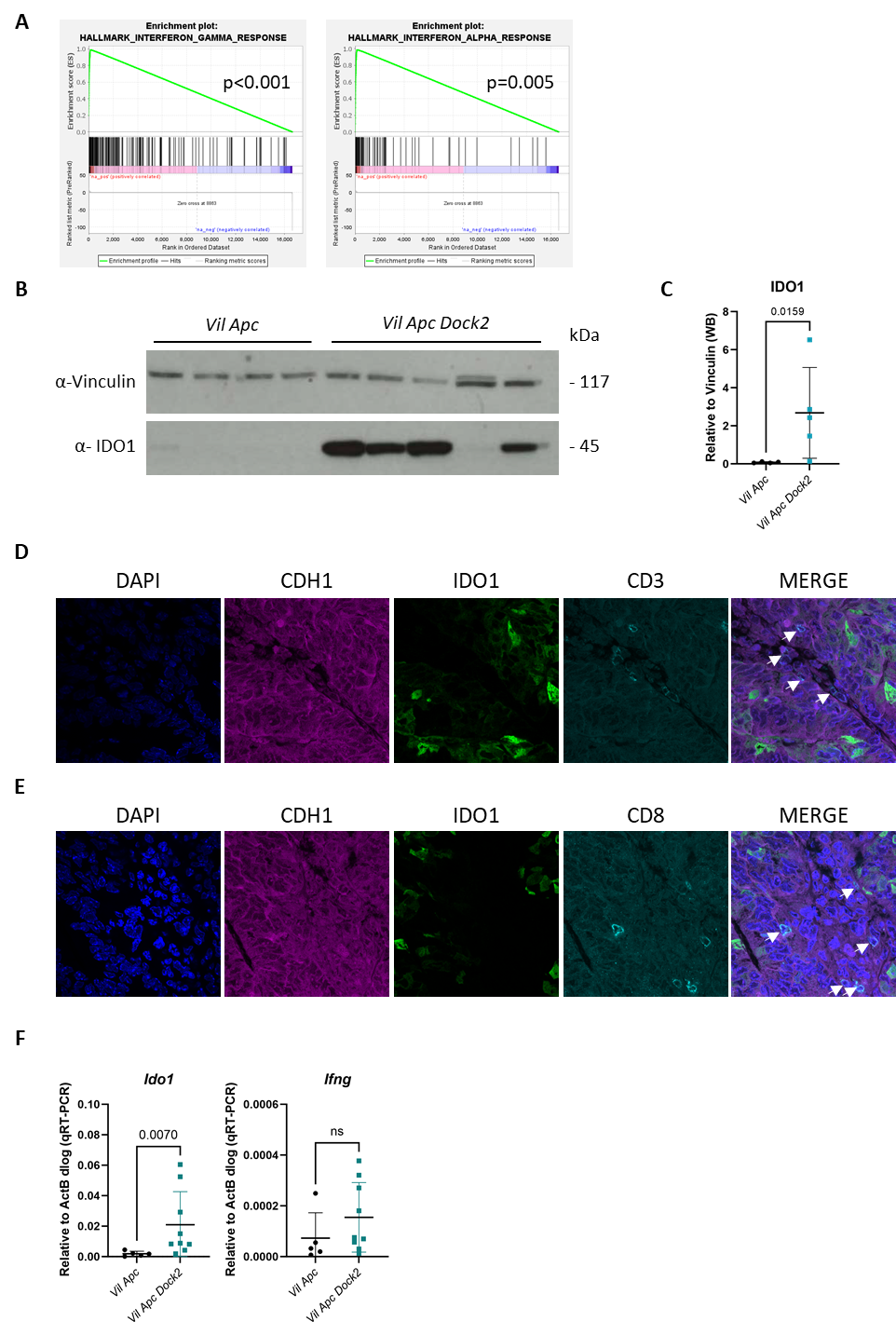
**

**Figure S2. *Dock2* deficient tumours have elevated IFNγ signalling.** (A) GSEA plots of IFNγ and IFNα response gene sets. (B) Western blot analysis of IDO1 expression in *Vil Apc* and *Vil Apc Dock2* tumours. (C) Quantification of IDO1 Western blot. N = 4 vs 5 tumours. (D) Co-immunofluorescence staining of *Vil Apc Dock2* tumour for Cdh1, Ido1 and CD3 expression. CD3+ cells are marked on the Merged panel demonstrating lack of co-expression with Ido1. (E) Co-immunofluorescence staining of *Vil Apc Dock2* tumour for Cdh1, Ido1 and CD8 expression. CD8+ cells are marked on the Merged panel demonstrating lack of co-expression with Ido1. (F) RT-qPCR analysis of IFNγ target gene expression in normal adjacent tissue from tumour bearing *Vil Apc* and *Vil Apc Dock2* mice. N = 5 vs 9 mouse colons. Data represented as mean and error bars SD. All statistical analysis for this figure was performed using two-tailed Mann-Whitney test. Exact p values are indicated in the panels.


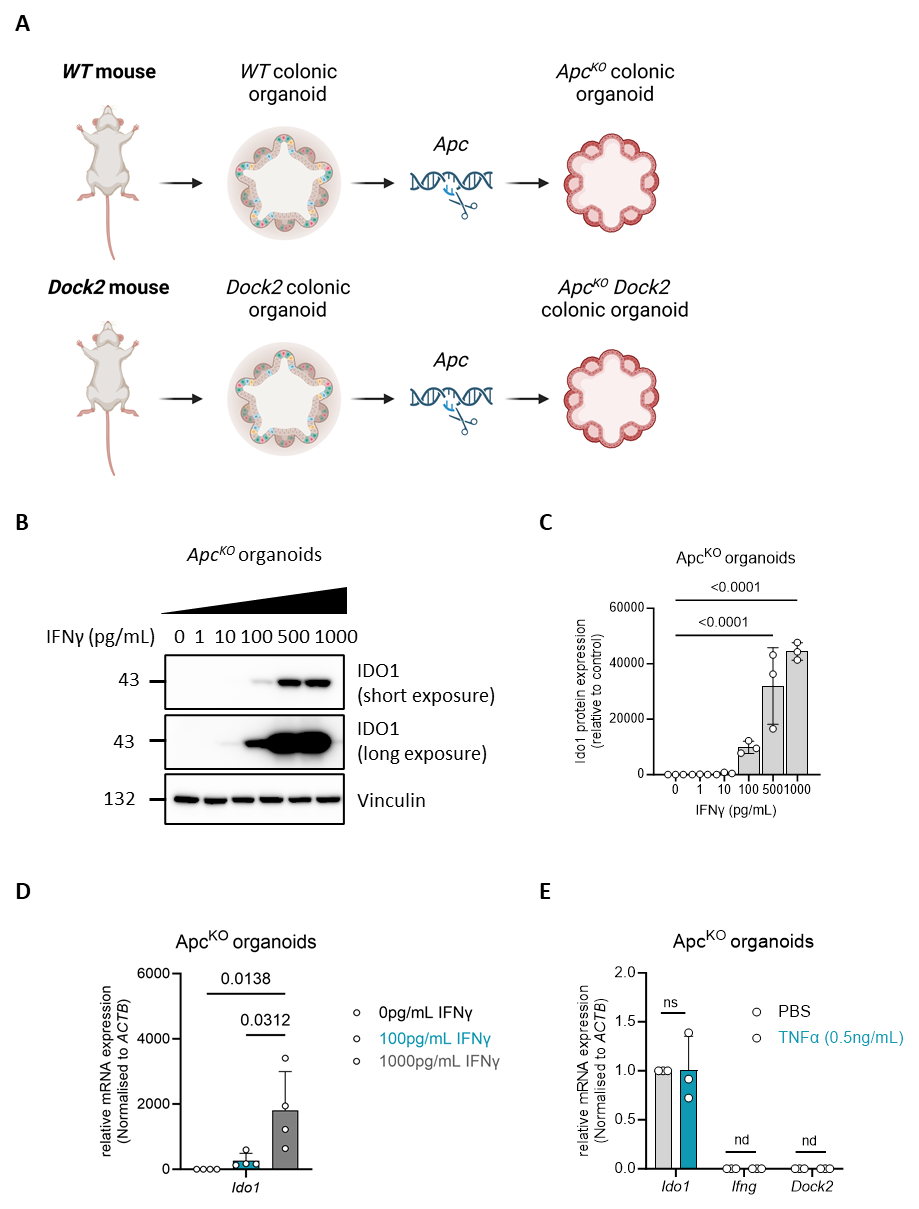


**Figure S3. IFNγ signalling is equivalent in normal and *Dock2* deficient tissue.** (A) Schematic outlining experimental strategy for deleting *Apc* in *Dock2* deficient colonic organoids. (B) Western blot analysis of IDO1 expression following treatment of *Apc^KO^* organoids with increasing concentrations of IFNγ. (C) Quantification of Western blot analysis. N = 3 independent technical replicates. (D) RT-qPCR analysis of *IDO1* expression following treatment of *Apc^KO^* organoids with increasing concentrations of IFNγ. N = 4 independent technical replicates. (E) RT-qPCR analysis of *Ido1, Ifng* and *Dock2* following treatment of *Apc^KO^* organoids with TNFα. Note nd = not detected. N = 3 independent technical replicates. Data represented as mean and error bars SD. Statistical analysis for (C), and (D) was performed using ordinary one-way ANOVA with Tukey’s multiple comparisons exact p values are shown.

**
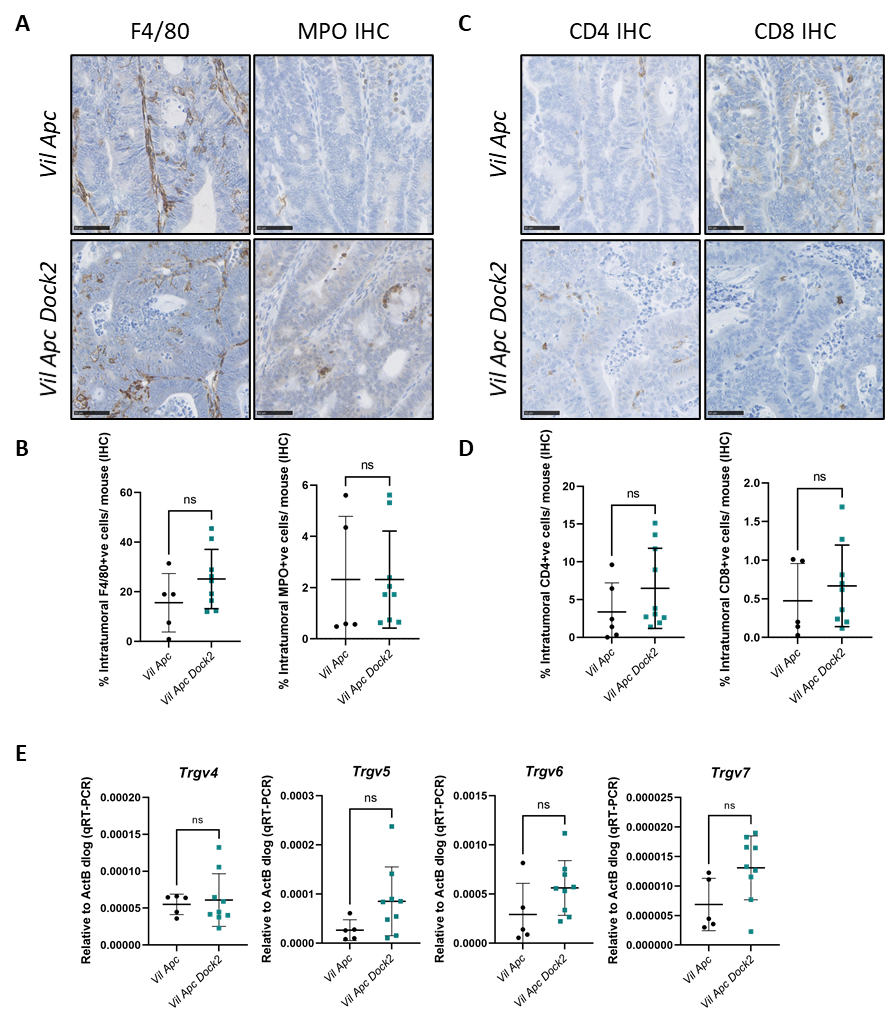
**

**Figure S4. *Dock2* deficient tumours have increased γδ T cell infiltration.** (A) Representative F4/80 and MPO staining of *Vil Apc* and *Vil Apc Dock2* tumours Scale bars are 50μm. (B) Quantification of F4/80 and MPO staining. N = 5 vs 9 mice. (C) Representative CD4 and CD8 staining of *Vil Apc* and *Vil Apc Dock2* tumours Scale bars are 50μm. (D) Quantification of CD4 and CD8 staining. N = 6 vs 10 mice (CD4) and 5 vs 9 mice (CD8). (E) RT-qPCR analysis of *Trgv4, Trgv5, Trgv6* and *Trgv7* expression in *Vil Apc* and *Vil Apc Dock2* tumours. N = 5 vs 9 tumours. Data represented as mean and error bars SD. All statistical analysis for this figure was performed using two-tailed Mann-Whitney test. Exact p values are indicated in the panels.

**
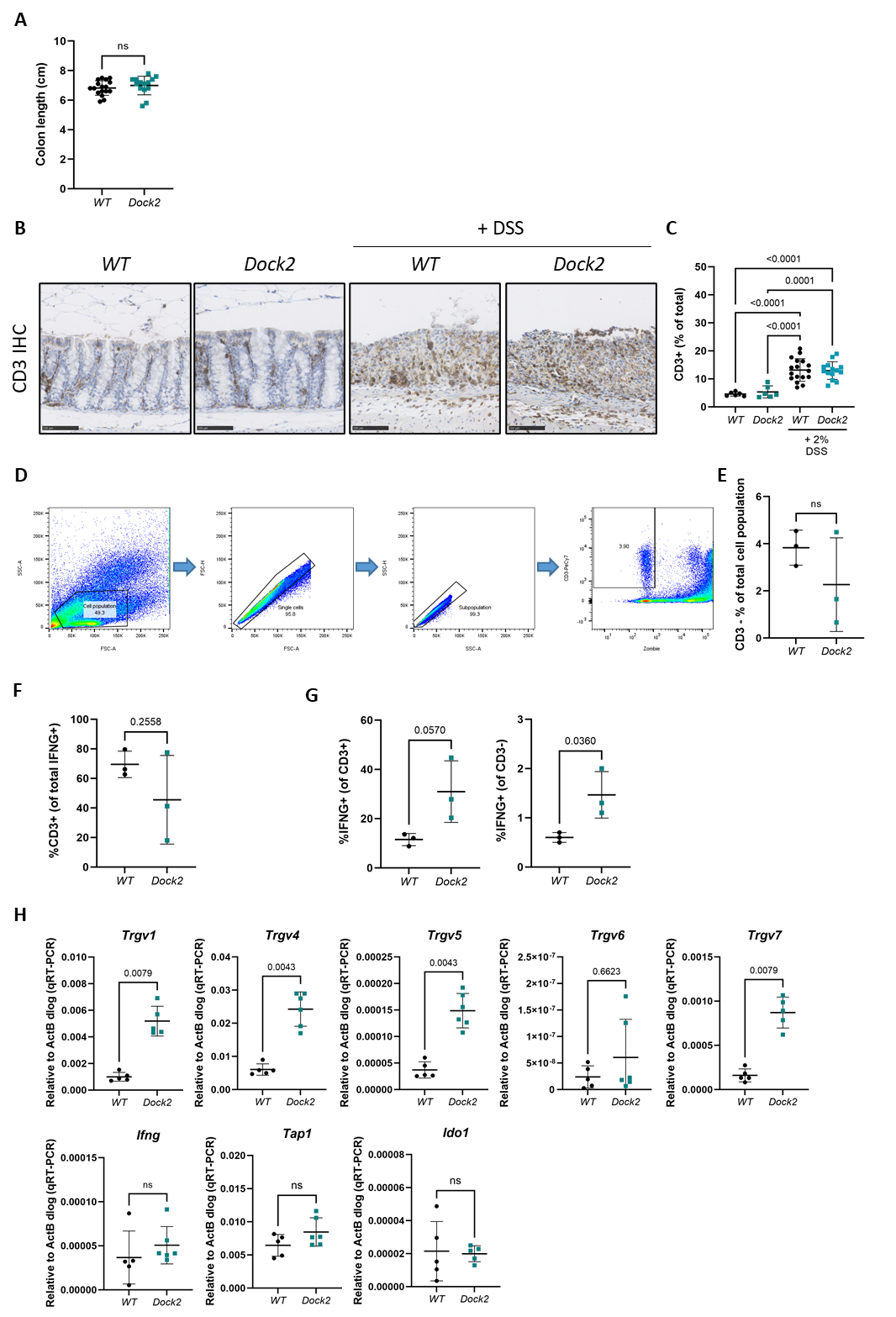
**

**Figure S5. IFNγ producing γδ T cells are increased in *Dock2* deficient colons independently of acute inflammation.** (A) Colon length of DSS treated *WT* and *Dock2* mice. N = 17 vs 14 mice. (B) Representative CD3-stained *WT* and *Dock2* colons at baseline or following acute DSS treatment. Scale bars are 100μm (C) Quantification of CD3 staining. N = 6 vs 6 vs 17 vs 14 mice. (D) Flow cytometry gating strategy (E) Quantification of CD3+ cells as a proportion of all colonic cells. N = 3 vs 3 mice. (F) Quantification of the percentage of cells expressing IFNG that are CD3+. N = 3 vs 3 mice. (G) Quantification of percentage of CD3+ cells that are IFNG+ (left panel) and percentage of CD3- cells that are IFNG+ (right panel) in colons from *WT* and *Dock2* mice. N = 3 vs 3 mice. (H) Expression of γδT cell markers and IFNγ target genes in thymus from *WT* and *Dock2* mice. N = 5 vs 5 mice (*Trgv1* and *Trgv7*), N = 5 vs 6 (all others). Data represented as mean and error bars SD. Statistical analysis for (A), (E), (F), (G) and (H) was performed using two-tailed Mann-Whitney test. Statistical analysis for (C) was performed using ordinary one-way ANOVA with Tukey’s multiple comparisons. Exact p values are indicated in the panels.


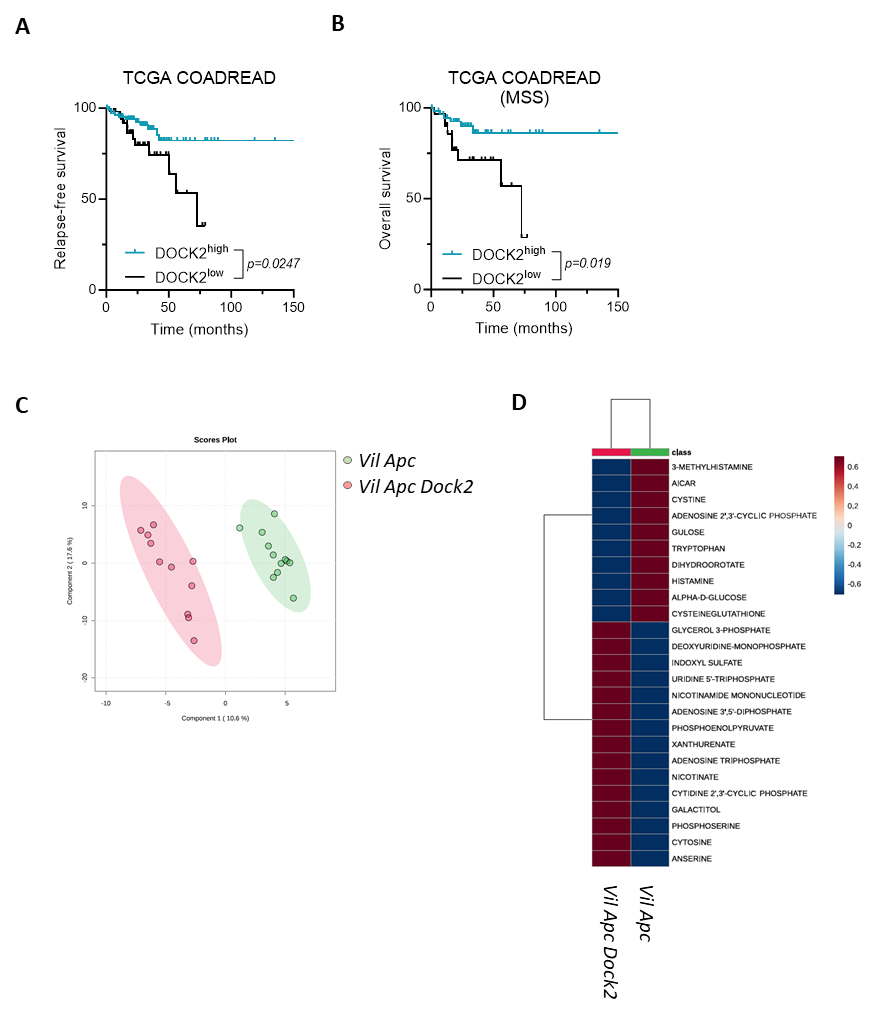


**Figure S6. IDO1 is elevated in human IBD-CRC and alters tryptophan metabolism *in vivo*.** (A) Survival analysis of entire TCGA CRC dataset separated for *DOCK2* expression. (B) Survival analysis of MSS TCGA CRC patients separated for *DOCK2* expression. (C) PLSDA scores plot of metabolomics data. *Vil Apc* tumours highlighted in green, *Vil Apc Dock2* tumours highlighted in red. (D) Hierarchical Clustering Heatmap showing only group averages using the TOP 25 metabolites according to their Variable Importance in the Projection (VIP) scores.

**
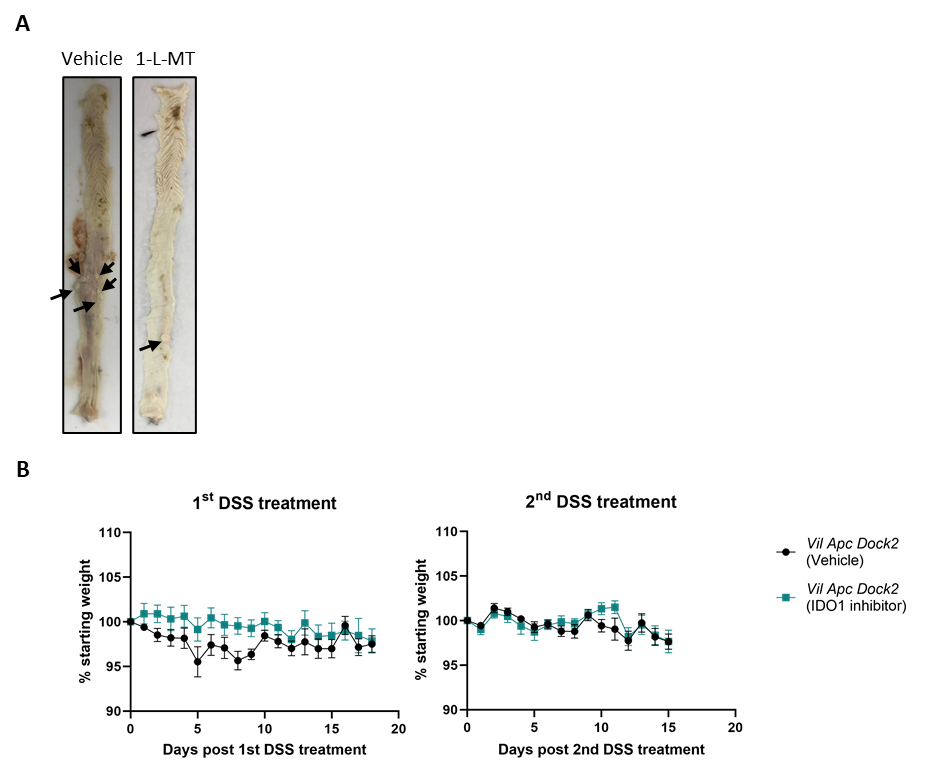
**

**Figure S7. IDO1 inhibition abrogates tumourigenesis in *Vil Apc Dock2* mice.** (A) Representative photographs of vehicle and IDO1 inhibitor treated *Vil Apc Dock2* mice at experiment endpoint. Tumours indicated with black arrows. (B) Comparative weights of vehicle and IDO1 inhibitor treated *Vil Apc Dock2* mice during DSS treatments. Data represented as mean and error bars SEM.

**Table S1. *Vil Apc* vs *Vil Apc Dock2* RNAseq.** RNAseq data comparing tumours from *Vil Apc* and *Vil Apc Dock2* mice.

**Table S2. Gene ontology analysis.** GProfiler gene ontology analysis of differentially expressed genes in *Vil Apc* vs *Vil Apc Dock2* tumours.

**Table S3. Metabolomic analysis.** Metabolomic analysis of *Vil Apc* vs *Vil Apc Dock2* tumours. Tumours labelled VADOK2 are *Vil Apc Dock2* and labelled VAL_APC are *Vil Apc.*

**Table S4. Primer sequences.** Primer sequences used for RT-qPCR analysis and CRISPR/Cas9 gene editing.
